# Sub-Diffraction Nano Manipulation Using STED AFM

**DOI:** 10.1371/journal.pone.0066608

**Published:** 2013-06-17

**Authors:** Jenu Varghese Chacko, Claudio Canale, Benjamin Harke, Alberto Diaspro

**Affiliations:** 1 Istituto Italiano di Tecnologia, Genova, Italy; 2 Department of Physics, University of Genova, Genova, Italy; University of Milano-Bicocca, Italy

## Abstract

In the last two decades, nano manipulation has been recognized as a potential tool of scientific interest especially in nanotechnology and nano-robotics. Contemporary optical microscopy (super resolution) techniques have also reached the nanometer scale resolution to visualize this and hence a combination of super resolution aided nano manipulation ineluctably gives a new perspective to the scenario. Here we demonstrate how specificity and rapid determination of structures provided by stimulated emission depletion (STED) microscope can aid another microscopic tool with capability of mechanical manoeuvring, like an atomic force microscope (AFM) to get topological information or to target nano scaled materials. We also give proof of principle on how high-resolution real time visualization can improve nano manipulation capability within a dense sample, and how STED-AFM is an optimal combination for this job. With these evidences, this article points to future precise nano dissections and maybe even to a nano-snooker game with an AFM tip and fluorospheres.

## Introduction

Integrated microscopic tools have played an important role in deriving multi dimensional know-how of a scientific scenario [1]. Microscopic methods evolve into hybrid modalities pouring out data sets in a single shot, thereby reducing sorts of instrumental drawbacks coming from recurring measurements on the same region of interest. It has been recently shown that such a combination of super resolution technique and scanning probe microscopy can provide complementary details on topology of specific structural details [2]. Combining an atomic force microscope (AFM) with light microscopy techniques like confocal microscopy has become a standard tool for various studies carried out using AFM [3] [4] and hence a step ahead to use a super resolution technique like STED with AFM is just in the path of evolution of nano scale techniques.

STED (Stimulated emission depletion) microscopy [5] [6] is a top contender in fluorescence based super resolution microscopy techniques. The key of STED is selectively switching off the fluorescence emission of the diffraction limited confocal focal volume in an intelligent manner [7]. By the principle of stimulated emission, a suitable wavelength chosen from the emission spectra of fluorescence can deplete the fluorescence with a finite probability. A complete depletion from the periphery of focal volume can be ensured by increasing the number of events, which is simply done by increasing the power in this donut-shaped depletion focus and thence gaining a higher resolution [8]. AFM (Atomic force microscopy) surpasses other microscopy techniques due to its simplicity in probing the sample. This technique studies the deflection of a soft cantilever caused by the interaction of a sharp stylus at the free end of the cantilever, availing inter-atomic forces. From the event of invention in 1986 [9], the technique has grown into a robust scientific method which can provide topography with nanometer resolution offering a capability to test local mechanical properties as well as intermolecular interactions, even in a controlled liquid environment [10] [11]. Although the first applications of the AFM were essentially related to imaging, the setup was soon exploited as a force sensor and as a nano manipulator [12]. In particular, AFM was used in the field of biology to characterize the mechanical properties of complex systems such as living cells, obtaining maps of local stiffness of the cell with a lateral resolution in the range of tens on nanometers [13]. In spite of the inherent aspecificity of the technique, AFM is also used for the study on large heterogeneous composition such as living cells. Fluorescence image provided by an optical microscope can be used to recognize particular components that is imaged by the AFM, but lost in the multitude of structures that compose the biological sample [14]. The method fluorescent tagging, super resolution imaging of the resultant structures and measurement of the force maps of an area of interest inside a eukaryotic cell was reported from our group [2].

Nanomanipulation is another capability of the AFM that has been explored in the last two decades[15]. Controlled positioning of nano particles, molecules or clusters of molecules is an important step towards the investigation of new class of chemical processes[16]. The handling of nanometer scale objects requires the non-trivial ability to recognize the object of interest, moving it and tracking it. AFM is one of the versatile techniques that approaches this goal, but limited by its unspecific nature and low operating speed. These problems can be overcome by enabling a real time feedback of the manipulation and AFM based methods like dynamic line scanning or contact- loss detection which provides an active feedback, thereby monitoring the movement[15] [17]. Although nano manipulation is a celebrated use of AFM in science and technology, AFM based nano manipulation is not always repeatable and reliable [18]. Hence, many other models, visualization routines and methods have been proposed in the recent years to estimate the interaction of the tip on the object, contemplate the movement and pursue the object [19]. But, a foolhardy way to do AFM based nano manipulation would be sequentially image in order to visualize the movement; i.e. coupling a optical technique that offers performance in terms of chemical recognition and speed can reach the same goal of pursuit, almost instantaneously. In this regard, STED is a beneficial replacement for current optical imaging schemes used with AFM, because it provides the imaging capability with a lateral resolution and high specificity in the order of tens of nanometers within a few seconds on large areas of interest [2]. In this article, we demonstrate how to use a STED microscope for assisting AFM to do imaging and manipulation with sub diffraction precision.

## Methods

### STED system settings

The laser beam from a Titanium Sapphire laser (Chameleon Ultra II, Coherent, Inc.) is fed into the scan box of the microscope (Nikon A1R MP, Nikon Instruments Inc), which is a multiphoton microscope by design. For making this beam into a STED beam, laser beam is rerouted through an optical geometry consisting of a 100 m single mode fibre, which stretches the pulse in time and a phase plate (RPC Photonics, Rochester) for phase engineering the beam to form a doughnut shaped PSF (Point Spread Function). Further circularly polarising the laser beam by a λ/4 quarter-wave plate ensures a proper doughnut shaped focus. A picosecond diode laser PDL-800D (637 nm PDL, Picoquant GmbH, Berlin) was used as the excitation laser, triggered by an electronic pulse from the multiphoton laser source. We used an oil immersion objective of high NA (Numerical Aperture) for measuring all the images (CFI Plan Apo VC 60X Oil, Nikon Instruments Inc). The resulting system was tuned for its depletion efficiency by looking at dye solution of Atto647N (Atto-tec GmbH, Siegen, Germany), suitable for the laser wavelengths we used: excitation (637 nm) and STED (760 nm).

### AFM Operation mode

**Figure 1:**
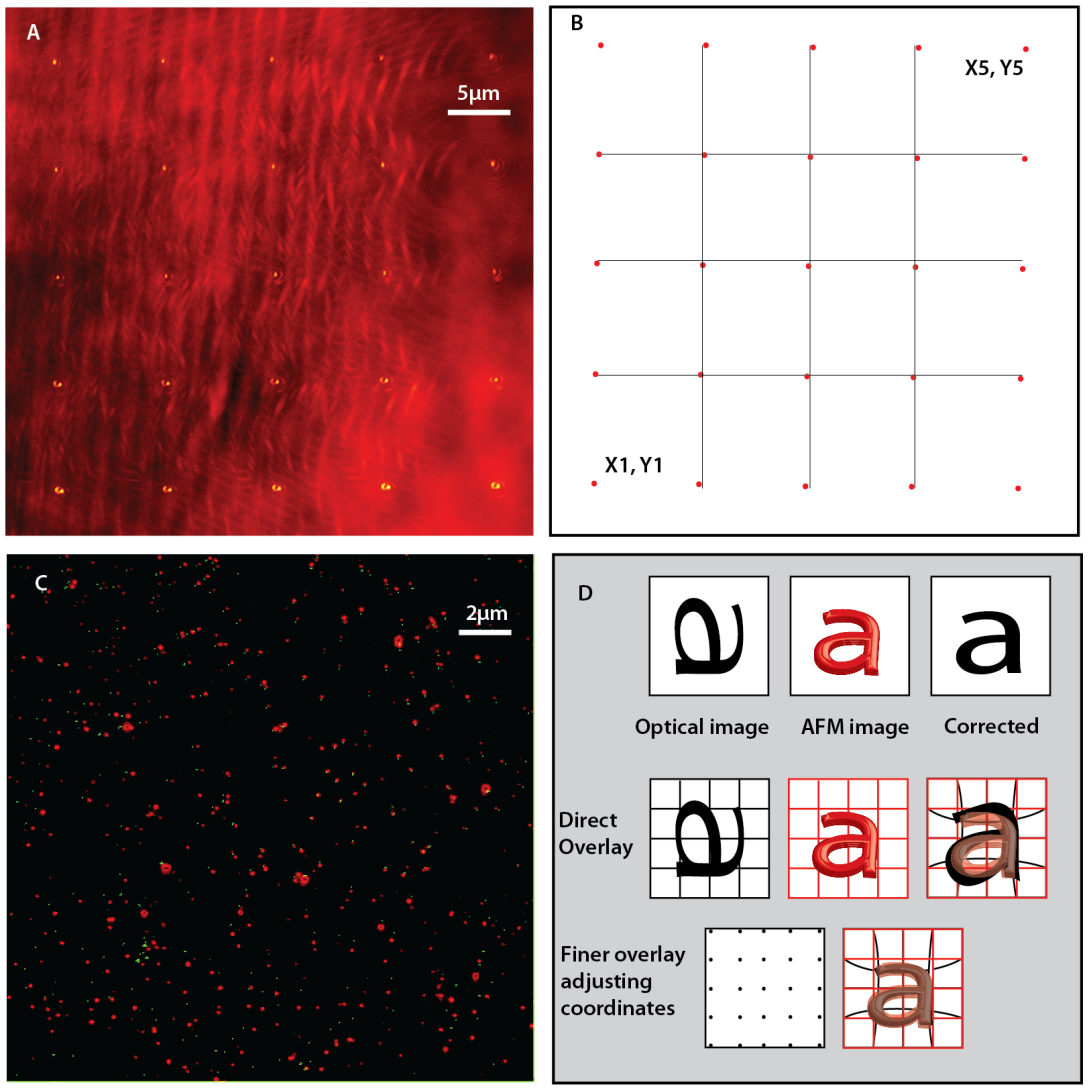
Fluorescence image overlay. The panel A shows the reflection images made on a 42×42 µm sq area of optical field of view by moving the tip in 35×35 µm sq in order to calibrate this area using 25 points as shown. This is a maximum intensity projection of 25 images to show their respective positions. The panel B shows how the grid is formed in the AFM software by a calibration. Panel C is an overlay of AFM on STED image using these calibration points given by AFM software. The green channel corresponds to AFM peaks while the red channel shows the STED image of the respective bead position. The overlay image is threshold corrected to enhance contrast. Although AFM software does a good job in overlaying confocal images, STED image overlay shows an imperfection, which gets stronger towards the borders. Panel D shows the total overlay procedure we follow where we edit the grid points to nonlinearly stretch and place the fluorescent image. In the first row of this panel, we show a cartoon of the collected optical image with inherent aberrations, AFM and the corrected image. Second row depicts the direct overlay software, which calibrates the pictures and makes a grid automatically. Nevertheless, a finer overlay is obtained by adjusting these grid points manually, especially when you overlay optical images from higher resolution imaging schemes where the pixel size of the image are well below the diffraction limit. Although the AFM software allows you to edit these values in its calibration ASCII file, we used a Labview routine for editing and artificially creating these set of 25 points.

An AFM setup (JPK Instruments AG, Berlin, Germany) was mounted on the above-mentioned microscope enabled with STED imaging. This procedure is made simple by the availability of suitable mountings for microscope models in the market; another benefit of using the commercial unit for building STED. The AFM was tested for noise isolation and calibrated on samples of known dimensions. In intermittent contact (IC) mode, the AFM tip is moved in a frequency regime close to the resonant frequency of the cantilever and the height information is extracted from the damping of the kinetic excitation signal. We have employed this modality of imaging for the topological images acquired in this article. Once the AFM is setup, we use the direct overlay option in the AFM software to visualize the fluorescent image as a map laid inside the AFM control software. This overlay requires a calibration procedure assisted by a grid of 25 points, which nonlinearly shifts, rotates and stretches the optical image to correct for all possible optical aberrations. The AFM software makes this grid from sequential reflection images of the tip’s actual movement in 25 piezo positions with a finite pixilation and scan size (Fig. 1A). The grid is shown for a 42×42 micrometer square area (Fig. 1B) and the respective overlay using direct overlay of AFM on 20×20 micrometer square area inside this calibrated area is visualized by the AFM software (Fig. 1C). For usual fluorescence images from wide field or confocal microscope, this grid gives good results, which matches the morphological features and has been successfully tested [14] [20]. However, employing a super-resolution imaging scheme, the resultant image seems realistically very difficult to match because of requirement of nano meter accuracy in the image overlay procedure and the presence of optical imperfections in the STED image in a large field of view. This can be manually fine-tuned by adjusting the grid points as shown in Fig. 1D to obtain a desired overlay. We used a Labview based program to arbitrarily read and write the calibration file, even without measuring the reflection image (Fig. 1A). Unwanted vibration of the sample is a performance limitation for AFM, hence for a better resolution of AFM, the objective is pulled back from the microscope to avoid any noise coupling through the objective immersion media used (water/oil).The measurements presented in this article are on fluorescent microspheres(Crimson, Invitrogen, USA) of sub-diffraction sizes.

## Results and Discussion

### Hybrid STED AFM setup

**Figure 2:**
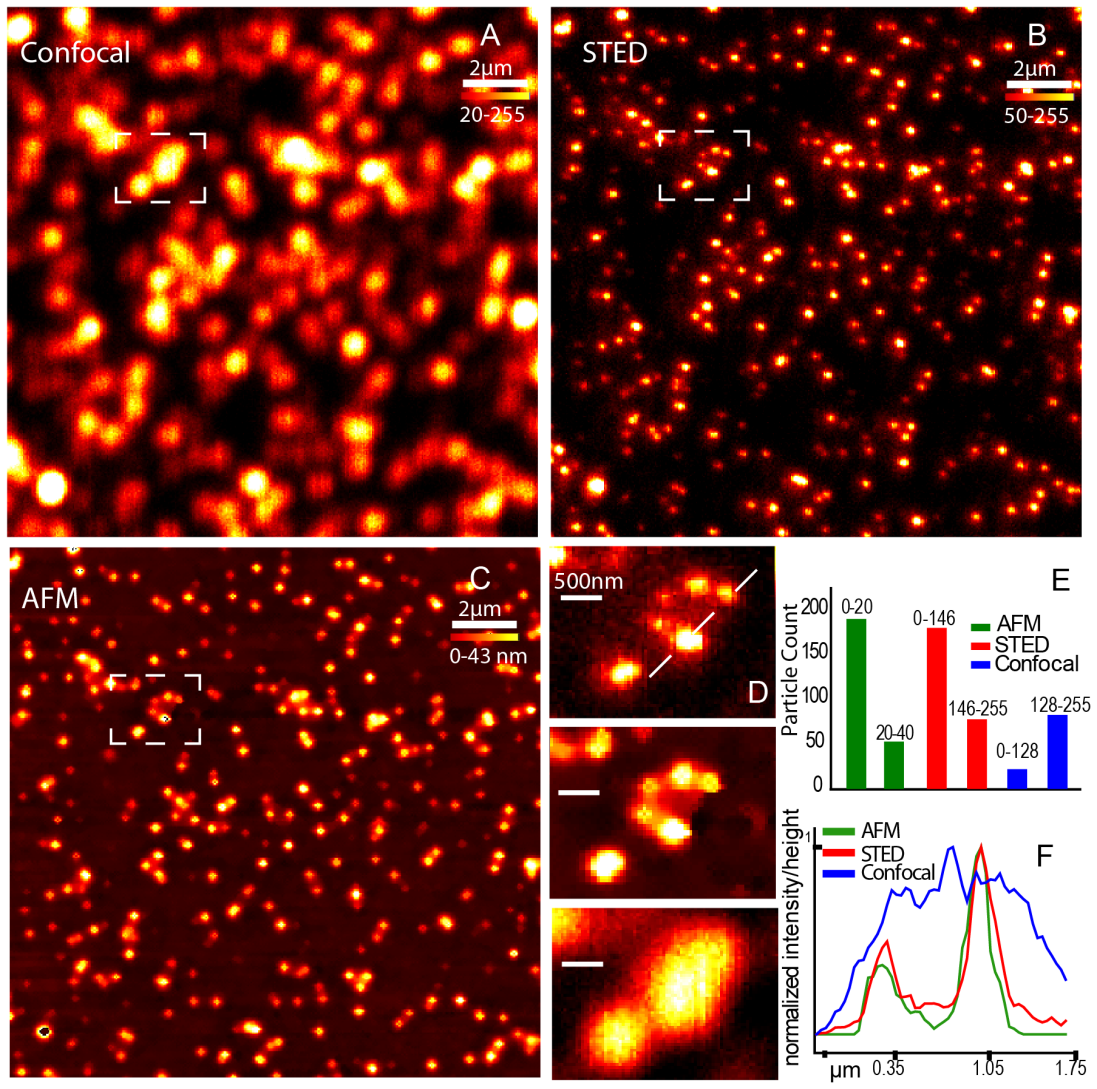
Quantitative STED imaging. A) Confocal, B) STED and C) AFM images of a sample consisting of a mixture of 20 and 40 nm beads fluorescing crimson. D) The insights also show the distinctive population identifiable in STED and AFM, while confocal is limited by diffraction. E) the graph shows the distinctive population ratio seen in AFM (green) and STED (red) both separated by a brightness based particle counting routine, while confocal is limited due to the high density of beads. F) The cross sectional line in the insights are plotted to show the resolution enhancement visualized in height image of AFM and brightness of STED while confocal brightness is blurred by diffraction.

To test how well the STED and AFM information couple, we used fluorescent beads of two different diameters placed on a glass cover slip to be sequentially imaged with Confocal, STED and AFM. We tested a mixture of 20 nm and 40 nm fluorescent spheres for this purpose because their sizes are below the diffraction limit. For AFM imaging in the IC mode, the information extracted is the relative height from substrate and in the case of spheres; it is same as the diameter of the object. STED and confocal images give fluorescent brightness information of an object and for fluorescent spheres; it corresponds to the diameter because the beads are homogenously filled with their respective dye molecules. In order to test how the information gained by these imaging modes match, a confocal image (figure 2A), a STED image (2B) and an AFM image (2C) of the same region of interest in a sample made from a mixture of 20 nm and 40 nm crimson fluorescent beads are cross-matched and verified in a quantifiable manner. Fluorescent images and AFM image were imaged with similar pixilation and the colour scale represents the relative height in the AFM image while it shows the relative fluorescent brightness in other two. We compare the lateral resolution with a closer look on a line profile (figure 2D) along the marked line (figure 2F). For through analysis of the data set, we used a particle counter routine in ImageJ to plot the size as two populations by choosing an appropriate threshold to divide the total numbers into two categories. This analysis routine use the colour scale values of the images and the prior knowledge that the sample has only two populations of objects. We ran this counting algorithm on the AFM image as well and plotted with STED and confocal population estimates. As elucidated in the graph (figure 2E), STED and AFM matches in their estimated numbers while the sample density hinders confocal counting algorithm. Confocal images could give the same information, if the sample concentration was low; which is not the case for cellular imaging where the fluorescent structures are crowded.

The STED image retains the quantitative information content and proven by this data, we suggest STED imaging as a lossless super resolution technique. The brightness of fluorescent structures usually correspond to the number of fluorescent molecules, and for a homogenous sample like the beads, it corresponds to volume or size of the structure. Inspecting the STED image helps to position the AFM tip to interesting regions from the necessary hints on the size and density of the sample of interest before approaching with the tip. On the other direction, STED by the inherent use of higher powers for higher resolution comes with the possibility of photo bleaching and the problems of fluorescence fluctuations in a sample, but a quantitative revaluation using AFM is a way to affirm any dataset. This is true for many correlational microscopy techniques, where one technique reinforce the observation of other’s mutually exclusive observation taking away the chances of instrumental fallacies.

### Nano manipulation

**Figure 3:**
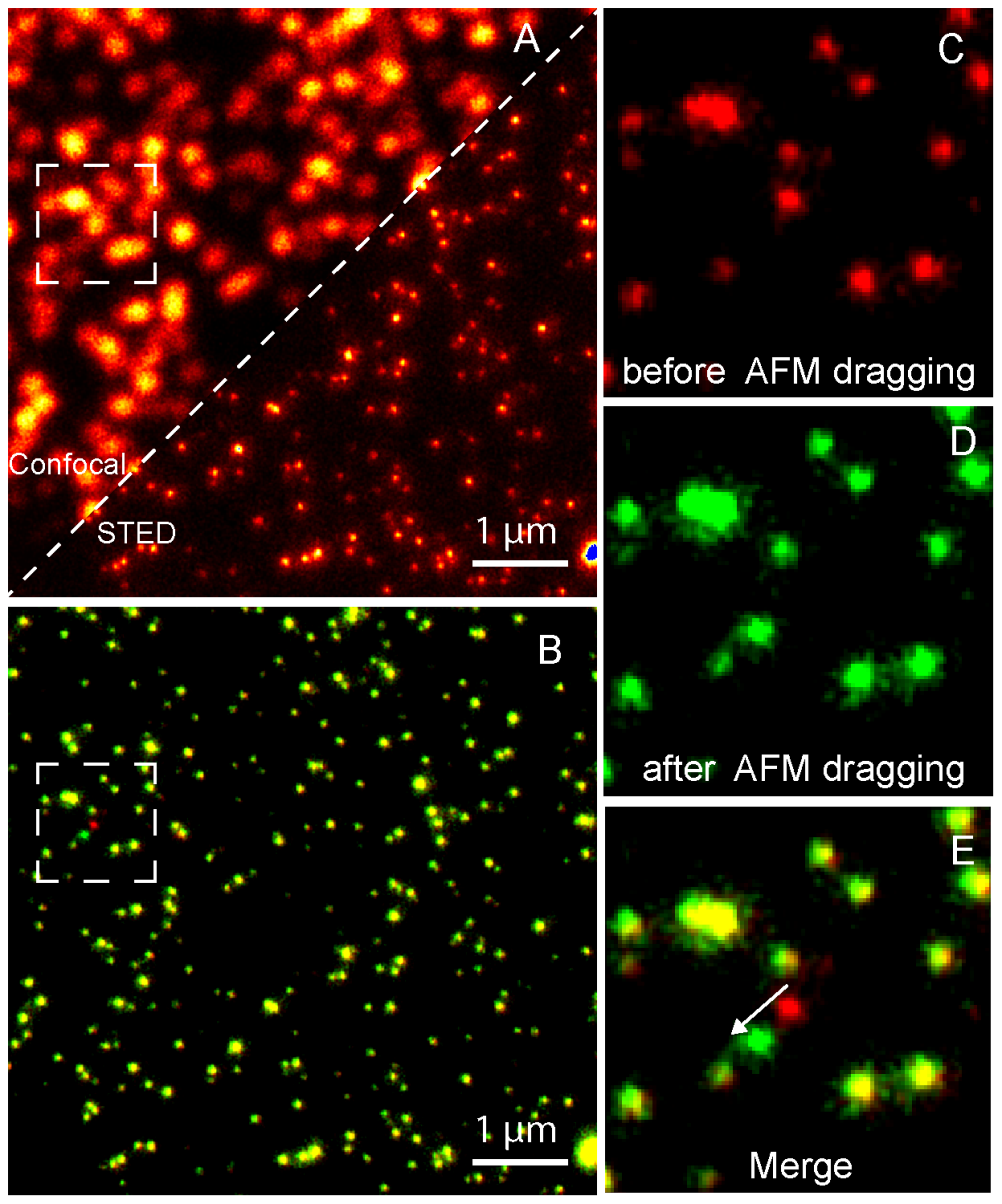
AFM Subdiffraction Nanomanipulation. (A) Confocal and STED images stitched in a single frame to visualize the resolution enhancement. (B) STED images done before and after AFM dragging at the same area are merged together in two colour channels. The red channel shows STED before AFM dragging and green channel shows after dragging. Hence, an overlay of colours shows stationary beads (yellowish). In this scheme, the single bead dragging can be visualized moving from red to green by the drag force. (C) A zoomed in crop of before AFM drag in red channel (D) shows after AFM drag in green channel. (E) Shows the merge of the images to emphasize the movement made is sub diffraction distance.

This above-mentioned result demonstrating the ability to see the objects with STED at a similar lateral resolution given by AFM motivated us to move to nano manipulation using STED AFM. With our instrument, we are able to tag and overlay AFM physical coordinates with STED fluorescent image. This sharing of coordinates suggests that by using the STED image as a map, we can precisely target the sample contents with the resolution capability offered by the STED image. We test this sub-diffraction targeting if the movement can be made on a single sub-diffraction sized target in sub-diffraction distance. We show in figure 3, diagonally overlaid consecutive images made on 40 nm crimson fluorescent beads with confocal and STED (figure 3A). We used the STED image as a map to direct the tip to any desired bead using the AFM software. Consequently, the AFM tip was commanded to move a single bead along in a desired path and we imaged the scene by STED before and after this dragging. These STED images are merged together in two colour scales for visualization (figure 3B). The red colour scale shows the STED image before AFM dragging and the green channel shows it after dragging. Hence, an overlay of colours shows stationary beads in yellow. In this scheme, the dragged single bead is seen as a bead in red, which has changed into green. A zoomed in crop of the area shows the beads before AFM drag in red channel (figure 3C), after AFM drag in green channel (figure 3D) and the merge of the images (figure 3E) to emphasize the movement made is sub diffraction distance. The rapid sub diffraction-imaging mode of STED microscope helps in doing such a measurement visualizes the high resolution targeting capability of the system. Notably, the AFM moved only one specific bead while all other beads remain in their position.

**Figure 4:**
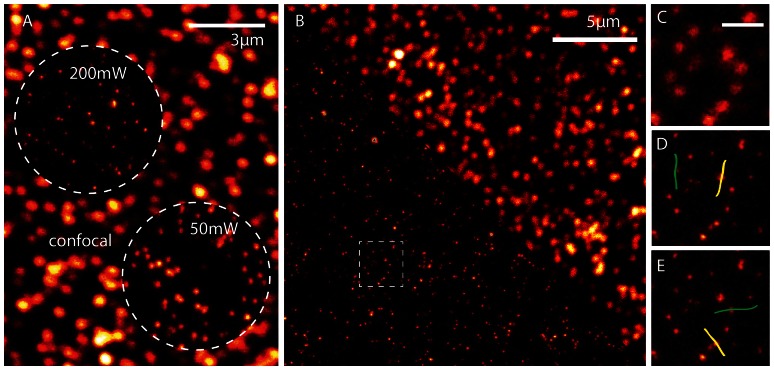
AFM Subdiffraction Nanomanipulation video. A) A confocal image of 40 nm beads is shown with two insights of two STED powers used in the context. The first insight with 200 mW STED power is used as the high-resolution map for AFM manipulation while a lower power of 50 mW was used for the video recording. The resolution regimes are evident in the image. B) Confocal and STED images stitched in a single frame to visualize the resolution enhancement. The marked area shows the area of manipulation. Figure C) shows a frame of the video to demonstrate the lower STED resolution we used for live recording inorder to avoid interaction of strong STED laser and tip. Panel D shows the marked lines in the AFM software for manipulation and imaging in the STED live imaging and panel E shows the marked lines for the confocal live imaging mode. We used the high-resolution image shown in panel B as the map for all the manipulation to ensure single bead movement and the live video for these four movements are shown in Video S1.

Nano manipulation in AFM was previously employed by using AFM images to assist the localization, tip handling and finally another AFM image to verify the movement [21]. It could take minutes of read out for an AFM, compared to STED image of same quality comes almost instantaneously. Since the lateral resolution of AFM is comparable to STED, with AFM STED, we are able to move ahead in this scenario and even visualize the dragging procedure itself in a fast manner. The results of this experiment are presented in a video (See Video S1) and explained with the help of figure 4. In order to avoid the AFM tip been exposed by strong STED light intensity and possible interactions, we reduced the STED power in this experiment. Figure 4A highlights the effect of reduced STED power used for live recording. If we perform a sequential imaging in STED and AFM modes instead of live monitoring, we could apply the full available power of 200 mW in the back aperture of the objective lens and reach the highest resolution (top left circle). However, for simultaneous use of both techniques, we reduced the STED power to 50 mW (bottom right circle). This interaction controlled STED imaging forces us to work at a slightly weaker resolution capability but ensures sub diffraction resolution live recording while the AFM tip is approached to the sample surface. In figure 4B, we show the STED/Confocal images to show the enhanced resolution image used for performing nano manipulation with the AFM. We concentrated on the marked area in figure 4B and we used the high-resolution STED data as the map and imaged it continuously in the lower power mode while AFM tip carried out the desired movements. Figure 4C shows the first frame of the video. In Video S1, we demonstrate movement of four beads. Two vertical dragging lines imaged in STED and two horizontal lines imaged in confocal mode. Illustrated in figure 4D and 4E, are the two manipulation line patterns drawn in the AFM software for each STED and Confocal imaging modes respectively. Labelled in yellow and green are lines manipulated in sequence at tip velocity of 100 nm/sec. As seen in the video, performing the experiment in the confocal mode makes the marked bead trajectories difficult to be identified. We manipulated different beads in the frame and moved them along the lines as indicated in the image. All other nearby fluorospheres in sub diffraction distances are not affected by the movement and this highlights the targeting precision is below the diffraction limit. The movements were precise in both STED and confocal because we used the high-resolution STED image for targeting and evidently, single beads are moved in all the four lines.

## Conclusion

We clearly show unambiguous visualization of STED and AFM of same area of interest in a sample of one to one correspondence in sub diffraction regimes. This method puts STED AFM in a position to do fast mapping of cells and materials before a detailed AFM probing. The resolution and specificity gained by the new setup enables to do AFM nano manipulation. Notably, the targeting precision demonstrated is below 40 nm, because we move single beads of this size without fail. This resulting tool can be seen as a nano scalpel, which can act as dissector, remover, or deliverer i.e. individual beads, can be separated, removed or delivered with careful practise. This set of proofs of principle give a new method to view experiments like learning assemblies, structural changes, etc or for cellular manipulation. Using two complementary microscopic techniques also enables to clarify sources of error in any scientific experiment. Genomics is another a field of interest provided an AFM tip can be used to rip out chromosomes to create DNA and chromosome band specific libraries[22] [23]. Our work suggests new capabilities in the interaction with nanometer-sized biomaterials and paves the way to new applications in the pioneer field of nano-surgery. A multi combination tool by adding a photon counting with AFM STED opens Fluorescence correlation spectroscopy (FCS) and AFM STED FCS, which indicates new potential in the investigation on membrane processes of an active species or following a protein aggregation pattern.

## Supporting Information

Video S1 **The Nanomanipulation video.** Two beads are moved in STED imaging mode and later in the confocal mode. The live recording was done at 5fps while the manipulation carried out at a 100 nm per sec in contact mode.
(AVI)

## References

[1] Smith C (2012) Microscopy: Two microscopes are better than one. Nature 492: 293–297. 23235883 10.1038/492293a

[2] Harke B, Chacko J, Canale C, Diaspro A (2012) A novel nanoscopic tool by combining AFM with STED microscopy. Optical Nanoscopy 1: 3 doi:10.1186/2192-2853-1-3

[3] Kassies R, Van der Werf KO, Lenferink A, Hunter CN, Olsen JD, et al (2005) Combined AFM and confocal fluorescence microscope for applications in bio-nanotechnology. Journal of microscopy 217: 109–116. 15655068 10.1111/j.0022-2720.2005.01428.x

[4] Dvorak JA (2003) The application of atomic force microscopy to the study of living vertebrate cells in culture. Methods 29: 86–96 doi:10.1016/S1046-2023(02)00284-0 12543074 10.1016/s1046-2023(02)00284-0

[5] Hell SW, Wichmann J (1994) Breaking the diffraction resolution limit by stimulated emission: stimulated-emission-depletion fluorescence microscopy. Optics letters 19: 780–782. 19844443 10.1364/ol.19.000780

[6] Hell SW (2009) Microscopy and its focal switch. Nature Methods 6: 24–32 doi:10.1038/nmeth.1291 19116611 10.1038/nmeth.1291

[7] Westphal V, Kastrup L, Hell SW (2003) Lateral resolution of 28 nm (λ/25) in far-field fluorescence microscopy. Applied Physics B: Lasers and Optics 77: 377–380.

[8] Harke B, Keller J, Ullal CK, Westphal V, Sch\önle A, et al (2008) Resolution scaling in STED microscopy. Opt Express 16: 4154–4162. 18542512 10.1364/oe.16.004154

[9] Binnig G, Quate CF, Gerber C (1986) Atomic force microscope. Physical review letters 56: 930–933. 10033323 10.1103/PhysRevLett.56.930

[10] Butt HJ, Cappella B, Kappl M (2005) Force measurements with the atomic force microscope: Technique, interpretation and applications. Surface Science Reports 59: 1–152.

[11] Diaspro A, Rolandi R (1997) Atomic force microscopy [Guest Editorial]. IEEE Engineering in Medicine and Biology Magazine 16: : 26 –27. doi:10.1109/MEMB.1997.582172. 10.1109/memb.1997.582172 9086368

[12] Sitti M (2001) Survey of nanomanipulation systems. Nanotechnology, 2001. IEEE-NANO 2001. Proceedings of the 2001 1st IEEE Conference on. pp. 75–80.

[13] Radmacher M, Cleveland JP, Fritz M, Hansma HG, Hansma PK (1994) Mapping interaction forces with the atomic force microscope. Biophys J 66: 2159–2165. 8075349 10.1016/S0006-3495(94)81011-2 PMC1275941

[14] Messa M, Canale C, Marconi E, Cingolani R, Salerno M, et al (2009) Growth cone 3-D morphology is modified by distinct micropatterned adhesion substrates. IEEE Trans Nanobioscience 8: 161–168 doi:10.1109/TNB.2009.2019109 19366649 10.1109/TNB.2009.2019109

[15] Requicha A (2008) Nanomanipulation with the atomic force microscope. Nanotechnology.

[16] Junno T, Deppert K, Montelius L, Samuelson L (1995) Controlled manipulation of nanoparticles with an atomic force microscope. Applied Physics Letters 66: 3627 doi:10.1063/1.113809

[17] Onal CD, Ozcan O, Sitti M (2011) Automated 2-D Nanoparticle Manipulation Using Atomic Force Microscopy. IEEE Transactions on Nanotechnology 10: 472–481 doi:10.1109/TNANO.2010.2047510

[18] Landolsi F, Ghorbel FH, Dabney JB (2012) Adhesion and Friction Coupling in Atomic Force Microscope-Based Nanopushing. J Dyn Sys, Meas, Control 135: 011002–011002 doi:10.1115/1.4006370

[19] Tafazzoli A, Pawashe C, Sitti M (2005) Atomic force microscope based two-dimensional assembly of micro/nanoparticles. The 6th IEEE International Symposium on Assembly and Task Planning: From Nano to Macro Assembly and Manufacturing, 2005. (ISATP 2005). pp. 230–235. doi:10.1109/ISATP.2005.1511478.

[20] Ludwig T, Kirmse R, Poole K (2007) Challenges and approaches—probing tumor cell invasion by Atomic Force Microscopy. Modern research and educational topics in microscopy no III 1: 11–22.

[21] Hansen LT, Kühle A, Sørensen AH, Bohr J, Lindelof PE (1999) A technique for positioning nanoparticles using an atomic force microscope. Nanotechnology 9: 337.

[22] Rubio-Sierra FJ, Heckl WM, Stark RW (2005) Nanomanipulation by Atomic Force Microscopy. Advanced Engineering Materials 7: 193–196 doi:10.1002/adem.200400174

[23] Fotiadis D, Scheuring S, Müller SA, Engel A, Müller DJ (2002) Imaging and manipulation of biological structures with the AFM. Micron 33: 385–397. 11814877 10.1016/s0968-4328(01)00026-9

